# Precision Medicine & Pharmacogenomics: Personalized Medication in Neuropsychiatric Disorders using AI and telepsychiatry

**DOI:** 10.1192/j.eurpsy.2022.1745

**Published:** 2022-09-01

**Authors:** N. Gkouvas

**Affiliations:** Hellenic Psychiatric Association, Board Member, Athens, Greece

**Keywords:** Precision Psychiatry, e-mental health, telepsychiatry, Artificial intelligence

## Abstract

**Introduction:**

The term “personalised therapy” refers to the use of genetic data to better treat or determine the predisposition to a specific genetic disease, with the ultimate goal of improving quality of life. Telepsychiatry and AI are key to support it..

**Objectives:**

Determine benefits of pharmacogenomic analysis (PGx) in CNS diseases regarding: - cost effectiveness - adverse drug reactions - reduced hospitalizations -drug interactions - efficacy - quality of life - “trial and error” approach avoidance

**Methods:**

Questionnaires before and after the treatment provided using PGX tests Telepsychiatry for consultation along face to face sessions were conducted. Artificial intelligence in data analyses

**Results:**

Benefits of pharmacogenomic analysis (PGx) in CNS diseases: - cost effective savings - prediction and prevention of adverse drug reactions - reduced hospitalization due to ineffectiveness of medication - reduced risk of drug interactions - more effective treatments - better quality of life for the patient - with the analysis (PGx) the “trial and error” approach is avoided

**Conclusions:**

In a number of studies in patients with mental disorders, pharmacogenomic analysis (PGx) has led to an increase in both clinical response and remission, better tolerated treatments, fewer side effects, and reduced treatment costs. In conclusion, pharmacogenomic analysis is ideal for patients with CNS diseases: a) Not responding to treatment b) Who in their history have many relapses and hospitalizations c) They show serious side effects d) Who do not comply with the treatment e) Taking many medications and suffering from serious illnesses f ) Who are wary of taking psychotropic drugs

**Disclosure:**

No significant relationships.

